# Effects of High Levels of Deoxynivalenol and Zearalenone on Growth Performance, and Hematological and Immunological Parameters in Pigs

**DOI:** 10.3390/toxins10030114

**Published:** 2018-03-07

**Authors:** Kondreddy Eswar Reddy, Jaeyong Song, Hyun-Jeong Lee, Minseok Kim, Dong-Wook Kim, Hyun Jung Jung, Bumseok Kim, Yookyung Lee, Dongjo Yu, Dong-Woon Kim, Young Kyoon Oh, Sung Dae Lee

**Affiliations:** 1 Animal Nutritional & Physiology Team, National Institute of Animal Science, Rural Development Administration, (55365)#1500 Kongjwipatjwi-ro, Iseo-myeon, Wanju 55365, Korea; dreswar4u@gmail.com (K.E.R.), jysong76@korea.kr (J.S.); hyunj68@korea.kr (H.-J.L.); mkim2276@korea.kr (M.K.); poultry98@korea.kr (D.-Wook.K.); hyjjung@korea.kr (H.J.J.); yoo3930@korea.kr (Y.L.); dwkim9405@korea.kr (D.-Woon.K.); oh665@korea.kr (Y.K.O.); 2 College of Veterinary Medicine, Chonbuk National University, Ilsan 54596, Korea; bskims@jbnu.ac.kr; 3 Swine Science Division, National Institute of Animal Science, RDA, Chungnam 31000, Korea; yudongjo@korea.kr

**Keywords:** deoxynivalenol, zearalenone, pig, toxin, antioxidant, serum, immune, diseases

## Abstract

*Background*: Deoxynivalenol (DON) and zearalenone (ZEN) are common food contaminants produced by *Fusarium* sp. Mycotoxins are a potential health hazard because of their toxicological effects on both humans and farmed animals. *Methods:* We analyzed three groups of pigs: a control group (fed a standard diet), and the DON and ZEN groups, fed a diet containing 8 mg/kg DON and 0.8 mg/kg ZEN respectively, for four weeks. *Results*: DON and ZEN exposure decreased body weight (BW), average daily feed intake (ADFI), food conversion rate (FCR), and the serum levels of immunoglobulin (Ig)G and IgM. The total antioxidant levels significantly decreased in serum and increased in urine samples of both treatment groups. Additionally, DON and ZEN exposure increased serotonin levels in urine. Hematological parameters were not affected by the investigated toxins. Microscopic lesions were evident in sections of kidneys from either treatment group: we found sporadic interstitial nephritis in the DON group and renal glomerulus atrophy in the ZEN group. The expression levels of inflammatory cytokines and chemokine marker genes were reduced in tissues from DON- and ZEN-exposed pigs. *Conclusions*: chronic ingestion of high doses of DON and ZEN alters the immune response and causes organs damage, and might be associated with various diseases in pigs.

## 1. Introduction

*Fusarium* species are widespread molds that produce different trichothecene mycotoxins, including deoxynivalenol (DON) and zearalenone (ZEN). They are frequently co-occurring and contaminate cereal-related foods, so *Fusarium* has the potential to adversely affect both human and animal health [1,2]. Pigs are regarded as the most susceptible species to these toxins, and contamination with *Fusarium* causes economic loss, mainly due to a decrease in food intake (and consequent body weight [BW]) and fertility, caused by DON and ZEN, respectively [3]. Exposure to DON and ZEN also causes an alteration of the immune cell function, dysregulation of the humoral immune response, and interference in the host resistance to pathogens [4]. Notably, the adverse effects of these toxins in pigs vary, based on their dietary concentrations. A few decades back, several studies have demonstrated cases of intoxication in pigs that were caused by grains contaminated with *Fusarium* mycotoxins [5]. 

DON affects the systemic immune response, as well as blood chemistry in growing pigs [6,7]. Dietary DON has been shown to increase total immunoglobulin (Ig)A titer in serum and to interrupt the function of dendritic cells in pigs [6,8]. A different study demonstrates that IgA, IgM, and IgG secretion is considerably altered in murine lymphocytes treated with DON [9]. DON also controls the specific immune response to ovalbumin (OVA) vaccination by enhancing the levels of IgA and IgG against OVA [10]. Low dietary concentrations (0.05–2.5 mg/kg feed) of DON are associated with decreased weight gain, anorexia, and immune changes, while acute higher concentrations induce hemorrhagic diarrhea, vomiting, and circulatory shock [6,11]. At the cellular level, one of the main problems caused by exposure to DON is the inhibition of protein synthesis through its binding to the ribosomes. Additionally, exposure to low levels of DON upregulates the expression of cytokines and inflammatory genes with simultaneous immune suppression, while high exposure induces leucocyte apoptosis and immune stimulation [12]. 

ZEN is a biologically potent toxic compound. The most commonly recognized effect of ZEN is its capability to bind to estrogen receptor and stimulate expression of estrogen responsive genes in a number of animal species, especially pigs [13,14]. ZEN stimulates intracellular oxidative stress that causes oxidative DNA damage and apoptosis [15,16]. Several studies have shown that ZEN and its metabolites have different effects on the innate immune system of pigs, and induce or suppress the expression of pro-inflammatory cytokines in peripheral blood cells. The same studies have demonstrated that ZEN has toxic effects on pig neutrophils and reduces IgG, IgM, and IgA levels, as well as tumor necrosis factor (TNF) synthesis in an in vitro model [17,18].

Importantly, the effects of DON and ZEN *Fusarium* toxins on pigs’ growth performance depend on the available source of purified or naturally contaminating mycotoxin in ingredients [19]. In pigs, these mycotoxins have adverse effects on DNA, RNA, and protein synthesis, and also cause lesions in various tissues. The chronic ingestion of a DON- and ZEN-contaminated diet induces significant histological changes on the liver, intestine, and lymphoid organs [20]. Prepuberal gilts fed diets contaminated with DON (2.1 to 9.57 mg/kg) and ZEN (0.004 to 0.358 mg/kg) show hepatocyte glycogen depletion, accumulation of hepatic interlobular connective tissue, and hemosiderosis in the spleen [21,22]. DON and ZEN are the most common contaminant of cereal crops, such as corn, wheat, oat, and barley. Therefore, the contamination with these toxins is an important food safety issue worldwide. About 98% of South Korea animal feeds were contaminated with both DON and ZEN mycotoxins [23,24]. According to Kim et al. [23], the current levels of DON in South Korea animal mixed grain feeds contain in the range of 32.8–950.25 ng/g, with the mean concentration of 353.32 ng/g. At the same time, Korea animal mixed grain feeds were contaminated with ZEN ranging from 1 to 932 µg/kg, with the mean of 70 µg/kg [24]. Based on Korean Food and Drug Administration, the levels of DON and ZEN in grains did not exceed the maximum acceptable limit 1 mg/kg and 132 µg/kg, respectively. According to survey on South Korea pig farms, pork producers are growing about 90% male pigs for pork production in their farms. Due to this reason, we have focused investigation only on male pigs for determination of the effect of higher doses DON and ZEN contamination in the feeds. 

The aim of this study was to examine, in pigs, the effect of DON and ZEN exposure on the growth rate, hematological parameters, organ weight, and immune function. Additionally, we investigated the effect of DON and ZEN on the expression of selected inflammatory cytokines, and determined the extent of the histological lesions they caused in the kidney. Our experimental model for chronic mycotoxicosis in pigs was generated upon ingestion of food highly contaminated with DON or ZEN (8 mg/kg and 0.8 mg/kg, respectively) for four weeks. There is the possibility of exposure within these higher ranges to pigs on farms, particularly in cases where the husbandry is very poor. Due to this reason we used higher concentrations of DON and ZEN in the present study. The effect of such high amounts of these toxins has not yet been investigated.

## 2. Results

### 2.1. Effect of DON and ZEN Exposure on Pig Growth Performance 

First, we examined the effects of DON and ZEN on the growth of pigs: Table 1 presents the growth performance data of pigs fed a diet containing the two toxins. The final average daily gain (ADG) of pigs fed a DON-contaminated diet was significantly lower than that of pigs fed a control and ZEN-contaminated diet (0.59 vs. 0.85 or 0.81 kg, respectively, *p* = 0.0001). One of the main hints of DON and ZEN exposure is reduced feed intake. The average daily feed intake (ADFI) for pigs fed contaminated DON and ZEN corn feed was slightly lower compared to control pigs (1.31 and 1.38 kg, respectively, vs 1.41 kg, *p* = 0.54), while the feed conversion rate (FCR) was significantly higher (*p* = 0.005) in the DON-exposed group than in the ZEN-exposed and control group (2.25 vs. 1.70 kg and 1.68 kg, respectively). Overall, pigs fed a DON-containing diet had significantly lower final body weight (BW) than those fed a ZEN-containing diet or control pigs (36.72 vs. 42.68 and 43.50 kg, respectively, *p* = 0.005) and had lower ADG, ADFI and higher FCR. We then evaluated, for liver and kidney, the ratio between the weight of the organ and that of the body, to detect possible organ toxicity. However, we did not find any significant change in the liver-to-body and kidney-to-body weight ratio. 

### 2.2. Effect of DON and ZEN Exposure on Hematological Variables

After pigs were fed for four weeks either the experimental or control diets, blood samples were collected to investigate the effects of DON and ZEN on hematological variables. As indicated in Table 2, DON and ZEN exposure did not significantly alter parameters, such as white blood cells (WBC), red blood cells (RBC), hemoglobin (HGB), hematocrit (HCT), mean corpuscular volume (MCV), mean corpuscular hemoglobin (MCH), mean corpuscular hemoglobin concentration (MCHC), platelets (PLT), mean plate volume (MPV), platelet distribution width (PDW), and procalcitonin (PCT). However, DON exposure was associated with a significant change in red blood cell distribution width-coefficient of variation (RDW-CV), and red blood cell distribution width-standard deviation (RDW-SD), which were higher than in the control group (*p* = 0.008 and *p* = 0.013, respectively).

### 2.3. Effect of DON and ZEN Exposure on Histopathology

**Figure 1:**
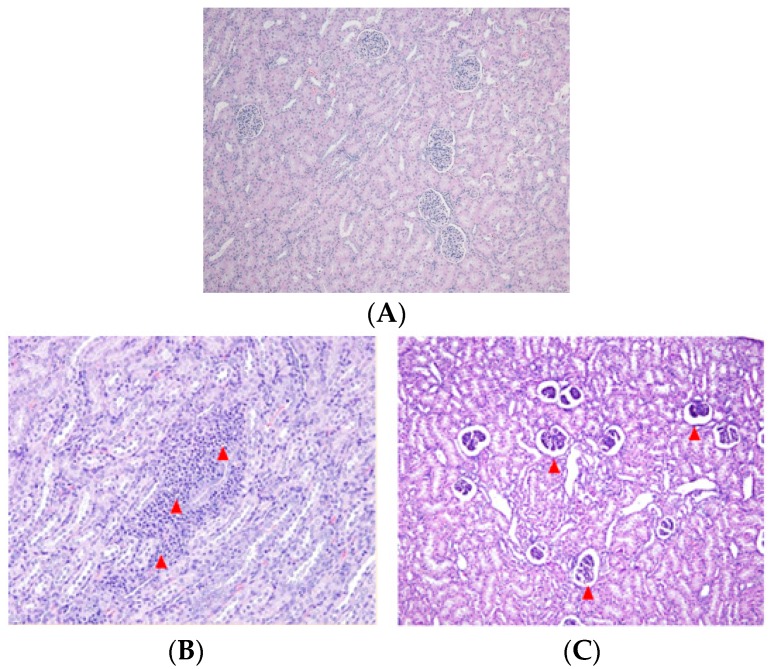
Histopathologic examination of the kidney of control and pigs exposed to deoxynivalenol- or zearalenone-contaminated diet for four weeks. (**A**) Kidney of pigs fed a standard diet (magnification, 100×); (**B**) Kidney of pigs fed a diet containing 8 mg/kg deoxynivalenol (DON). Infiltration of mononuclear inflammatory cell (arrowheads) was observed in the interstitial area (magnification, 200×); (**C**) Kidney of pigs fed a diet containing 0.8 mg/kg zearalenone (ZEN). Severe glomerulus atrophy (arrowheads) was noted (magnification, 100×).

Microscopic lesions were differentially evident in the kidney of DON- and ZEN-treated groups. No changes were observed in the kidney tissue of control group (Figure 1A). Sporadic interstitial nephritis consisting of mononuclear lymphocytes was noted in the DON-treated group (Figure 1B). Severe renal glomerulus atrophy was noted in the ZEN-treated group (Figure 1C). Prominent lesions were not observed in liver, duodenum, jejunum, ileum, caecum, and colon tissues from either group (data not shown).

### 2.4. Effect of DON and ZEN Exposure on Immunological Variables

After pigs were fed the experimental or control diets for four weeks, we examined the effect of DON and ZEN exposure on the immune response of the pigs. For this purpose, we measured the levels of various immunoglobulin subsets in the serum. As shown in Table 3, DON and ZEN significantly affected the serum levels of IgG (DON, 5.97 mg/mL; ZEN, 6.72 mg/mL; control, 8.46 mg/mL; *p* = 0.0001) and IgM (DON, 1.08 mg/mL; ZEN 1.24 mg/mL; control, 1.84 mg/mL; *p* = 0.0001). By contrast, DON and ZEN did not significantly affect the levels of IgA.

### 2.5. Effect of DON and ZEN Exposure on Total Antioxidant Capacity and Serotonin Levels

We also monitored the effect of DON and ZEN on the total antioxidant content (TAC) and serotonin levels, both in serum and urine samples, after pigs were fed for four weeks with the experimental or control diets. As shown in Table 3, TAC levels were significantly decreased (*p* = 0.045) in serum samples of pigs fed with DON- and ZEN-contaminated food, compared with control (88.24 and 95.98 vs. 109.45 nmol). Additionally, the TAC levels were significantly higher (*p* = 0.007) in the urine of DON-exposed pigs (4.33 nmol) than in that of ZEN-exposed pigs (2.40 nmol) or in the control group (2.36 nmol). Similarly, the levels of serotonin in the urine of DON- and ZEN-fed pigs (0.17 and 0.16 μg/mL, respectively) were significantly (*p* = 0.020) higher compared with the control group (0.08 μg/mL). 

### 2.6. Effect of DON and ZEN on the Expression of Immune Response-Related Genes in Muscle, Liver, and Kidney

To evaluate the defense mechanisms of different organs against DON and ZEN exposure, we quantified the mRNA expression of genes related to inflammatory cytokines. Table 4 shows the relative expression of six inflammatory genes (interferon gamma (IFN-γ), interleukin (IL)6, IL10, IL12B, prostaglandin-endoperoxide synthase 2 (PTGS2), and tumor necrosis factor-α (TNF-α)), three chemokines (C-X-C motif chemokine ligand 10 (CXCL10), chemokine (C–C motif) ligand (CCL)4 and CCL2) and one epithelial defense gene (claudin 3 (CLDN3)) in muscle, liver, and kidney samples of pigs exposed to DON or ZEN. We found that the expression of some of these genes, especially that of cytokines, varied considerably upon exposure to the mycotoxins in a tissue-specific way. When compared with the control group, DON treatment caused a significant increase in the expression of IFN-γ, IL6, IL12B, TNF-α, PTGS2, and CCL2 genes in the muscle, while the expression the same genes significantly decreased in muscle of the ZEN-treated group. Exposure to either DON or ZEN caused significant suppression of IFN-γ, IL10, IL12B, CCL4, and CLDN3 in the liver samples, compared with the control treatment. Additionally, DON and ZEN significantly decreased the expression of IL6, CCL4, and CLDN3 in the kidney (Table 4).

## 3. Discussion

In the present study, we investigated the effects of the exposure of pigs to the mycotoxins DON and ZEN. We found that the ADG and BW were negatively affected by DON exposure (compared with the ZEN and control groups). At the same time the FCR was higher in the DON dietary group (2.25 kg) than ZEN (1.70 kg) and control (1.68 kg). Importantly, we found decreased feed consumption, especially in the DON-exposed pigs. Our results agree with previously published data reporting that DON and ZEN ingestion negatively affects feed intake, and is consequently associated with a decrease in weight gain in the growing pigs and an increase in the FCR [25,26,27,28]. However, we found that exposure to DON had a more pronounced effect than ZEN exposure. According to Rotter et al. [4], contamination with higher levels of mycotoxins can lead to whole feed rejection and vomiting. During the treatment period, we observed that both DON- and ZEN-exposed pigs had diarrhea (data not shown), similarly to what was earlier reported by Bergsjø et al. [29]. 

A study from Chattopadhyay et al. [30] has shown that mycotoxins are deposited in the liver and kidney of BALB/c mice, causing damage and increased relative weight in these organs. However, in this study, we did not observe any significant effect of DON and ZEN on organ weight, but noticed small injuries in the kidneys of DON and ZEN dietary groups. An earlier study shows that the comparative weight of liver and kidney increases, but no lesions were observed in their histopathological evaluation of Sprague-Dawley rats gavaged with DON [31]. Contrarily, according to Xiao et al. [32], DON-exposed pigs show injury and higher weight of the kidney, and observed minor injury, but no significant difference in the weight of the liver. We believe that these inconsistencies are due to differences in pig age, dietary concentration of DON and ZEN, treatment period, and diet composition. 

Very limited information is available regarding the hematological effects of DON and ZEN exposure in pigs [33]. In the present study, we only found a significant effect of DON on RDW-CV and RDW-SD. However, we observed a slight decrease in RBC and HGB content and white blood cells, and a slight increase in the WBC and PLT count in both experimental groups compared with control (Table 3). Decreased RBC and HGB in pigs upon DON exposure have been shown by Zhao et al. [34]. Similarly, a study from Rotter et al. [35] measured different hematological variables in pigs fed increasing concentrations of DON (750, 1500, and 3000 µg/kg feed) and found a corresponding increase in the number of total leukocytes (10.3%, 8.7%, and 31%, respectively), which was mostly due to an increase in the number of segmented neutrophils. Limited information related to changes in RDW upon toxin exposure is available. In the present study, RDW was increased in the DON-exposed group compared with the ZEN-exposed and control groups. RDW is a parameter related to red blood cell volume and size; larger red blood cells might indicate a problem, and changes in the size of red blood cells are associated with several dysfunctions, such as sickle cell disease, malnutrition, chronic liver disease, and dimorphic anemia. Similar to our results, a study found that the RDW was significantly higher in DON-exposed animals [36]. 

To our knowledge, very little information is available on the effect of mycotoxins on the histopathology of pig organs. We found sporadic interstitial nephritis, indicated by the presence of mononuclear lymphocytes, in the kidney of pigs fed a DON-contaminated diet, and severe renal glomerulus atrophy in the ZEN-exposed pigs. We hypothesize that the high amount of DON and ZEN in the feed induced severe renal lesions. Similarly, an in vivo study from Tiemann et al. [22] demonstrates that pigs fed a diet contaminated with both DON and ZEN showed histological changes in their livers. 

The immune system is very sensitive to DON and ZEN exposure [12,18]. Attempts to estimate the effect of DON and ZEN on pig humoral immune response have provided conflicting results. An IgA raise after dietary DON treatment was found by Swamy et al. [7], Drochner et al. [33], and Goyarts et al. [37], but at the same time, other studies failed to show the connection between DON exposure and IgA levels in the serum [7,29,38]. According to Accensi et al. [6], there is no change in IgA, IgG, and IgM production after feeding pigs with 280, 560, and 840 µg/kg of a DON-contaminated diet for four weeks. In our study, the levels of IgG and IgM in the serum of pigs fed DON- and ZEN-contaminated food were significantly impaired, while we did not observe any effect on IgA levels. Our results are consistent with the results from Goyarts et al. [37], showing no significant difference in serum IgA levels between control and DON-treated pigs and a significant effect on IgG and IgM levels. Partially similar results are shown in a study by Atroshi et al. [39], showing a significant effect of a DON-polluted diet (6.25 mg/kg BW for one week) on IgG, IgM, and IgA levels. A different study indicates that DON (2.5 mg/kg BW) and ZEN (20 and 30 mg/kg BW) exposure for two weeks is associated with increased serum IgA and IgG levels, and decreased IgM levels [40]. Few studies have examined the changes in the immune response in pigs exposed to ZEN. According to Marin et al. [18], ZEN-treated pig peripheral blood mononuclear cell shows a significant decrease in IgG, IgM, and IgA levels at ZEN concentrations higher than 5 µM. DON and ZEN might suppress humoral immunity. According to a study from Pestka [31], the high concentration of DON and ZEN might decrease the serum IgG levels by promoting the apoptosis of T and B lymphocytes. Similarly, the study from Geisberger et al. [41] indicates that DON and ZEN induce the apoptosis of B lymphocytes, which secrete IgM. 

The connection between exposure to *Fusarium* toxins and the antioxidant status of pigs is unclear. In the present study, we observed that the TAC levels significantly decrease in the serum and increase in the urine of pigs fed DON- and ZEN-contaminated feed. Earlier studies have shown that either DON or ZEN might cause oxidative damage in in vitro and in vivo studies. Ren et al. [42] showed that DON triggers oxidative stress and induces cytotoxicity in splenic chicken lymphocytes in vitro. In the current study, because of the acute toxicity of DON and ZEN, we found antioxidants in the urine of the treatment groups. Similarly, the serotonin levels decrease in the serum and increase in the urine samples. According to Swamy et al. [7], when pigs consume high concentrations of mycotoxins, symptoms include decreased growth rate, vomiting, weariness, and alterations to the hypothalamus, including elevation of serotonin. Notably, serotonin is involved in the regulation of mood and anxiety, so low brain serotonin levels could contribute to augmented anxiety and depression [43]. 

In the present study, the most obvious effect of DON and ZEN exposure was observed on the expression of inflammatory and chemokine genes. We found a downregulation of some of the inflammatory genes in muscle, liver, and kidney, indicating the inhibition of the immune system. Similarly, a study has found that the expression of pro-inflammatory genes is downregulated in blood and ileum samples of DON-exposed pigs [44], and a different study indicated that the levels of pro- and anti-inflammatory cytokines dramatically decrease in the liver of pigs exposed to ZEN [45]. Other studies have shown different results. A study shows that DON food contamination (3 mg/kg) upregulates the expression of pro-inflammatory genes in the jejunum and ileum [46]. Similarly, a different group has found that ZEN-fed pigs (3.16 mg/kg) show elevated levels of inflammatory molecules in the spleen [47]. These differences might be caused by the different treatment regimens applied, treatment period, animal age, and other factors, which, in turn, might have an effect on the immune response. DON and ZEN are immunomodulators, and can therefore decrease or increase the production of inflammatory cytokines in the muscle, liver, and kidney. We hypothesize that DON and ZEN might have an effect in vivo through the suppression of the inflammatory response in muscle, liver, and kidney. DON and ZEN decreased the expression of IFN-γ and chemokines, which play a major role in the host defense against intracellular infections. Similarly, the two toxins decrease the expression of innate immune response genes, such as TNF-α and IL6, in the muscle and liver. A study from Abid-Essefi et al. [48], reported that the genotoxic effect of ZEN is due to the DNA fragmentation it causes, which leads to inhibition of protein synthesis. Additionally, a different study found that ZEN and its derivatives suppress the expressions of pro-inflammatory cytokines and induce DNA fragmentation [49]. Notably, DNA fragmentation is one of the events associated with the toxicity of ZEN in human hepatocarcinoma cells [49]. We found a decrease in the levels of some inflammatory cytokines in the liver and kidney of DON- and ZEN-exposed pigs; these organs, according to several studies, are more sensitive than the muscle. It is also known that deficient supply of essential macro- and micronutrients suppresses the immune response. According to Cunningham-Rundles et al. [50], malnutrition inhibits the release of pro-inflammatory cytokines. Therefore, it may also possible that the reduced expression of the inflammatory cytokines we noticed is due an indirect effect of DON and ZEN on the feeding of the pigs. 

## 4. Conclusions

The current study indicates that exposure to DON negatively affects the growth of pigs mainly by reducing the feed intake. We found changes in IgG and IgM levels, organ damage, reduced antioxidant capacity and serotonin levels in both serum and urine samples of DON- and ZEN-exposed pigs. DON and ZEN also caused hepatic and renal immune suppression by significantly reducing the expression of some of the inflammatory cytokines; microscopic lesions were accordingly observed in the kidney. Therefore, these toxins might have a significant role during infection. Because DON and ZEN are regular pollutants of animal food, the outcomes of this study might contribute to the establishment of more stringent limits of tolerance for these toxins in feeds for pigs.

## 5. Materials and Methods 

### 5.1. Ethics Statement

The protocols used for the animal experimental procedures were reviewed and approved by the Institutional Animal Care and Use Committee of the National Institute of Animal Science, South Korea No. 2015-147 of 29 May 2015. 

### 5.2. Animal Exposure to DON and ZEN and Experimental Design

The present study was carried out with 15 castrated males, 6 week old growing pigs with an initial average BW of ~19 kg. Each pig was housed in a separate pen (2.1 m × 1.4 m), at 25 ± 1 °C, with free access to water from drinking nipples, and was fed individually. The pigs were first allowed to adjust for one week to their new housing conditions and then divided into three treatment groups (*n* = 5 for each group), with equal BW. The control group was fed a standard diet (Table 5) [51], while the two treatment groups were fed a standard diet plus DON (8 mg/kg) or ZEN (0.8 mg/kg) for four weeks. DON and ZEN were purchased as purified toxins (Biomin Pte. Ltd., Singapore). During the entire experimental period, food and water were supplied *ad libitum*. 

### 5.3. Mycotoxin Analysis

Quantitative determination of DON and ZEN in DON and ZEN-contaminated corn feeds was performed using ultra performance liquid chromatography (UPLC). Homogenized DON- or ZEN-mixed grain samples (1 g) were extracted with 20 mL of distilled water and shaken for 30 min (DON samples) or with 0.5 g of NaCl and 20 mL acetonitrile (ACN) and shaken for 1 h. After filtering the extract through Whatman paper (No. 1), 5 mL of the DON filtrate sample were diluted in 20 mL of phosphate-buffered saline (PBS), and 5 mL of the ZEN filtrate sample were diluted in 20 mL of 1% Tween 20 solution. The diluted DON and ZEN samples were loaded separately onto immunoaffinity chromatography (IAC) columns. The DON columns were allowed to dry and then washed with 10 mL of PBS, 10 mL of distilled water, and then eluted with 0.5 mL of MeOH and 1.5 mL of ACN. In the case of ZEN, the column was washed with 10 mL of distilled water, and eluted with 1.5 mL of MeOH. Elutes were dried under N_2_ gas and 10 µL of each sample injected into a UPLC instrument (Water Acquity UPLC^®^ H Class, Milford, MA, USA). The mobile phase used to separate the DON and ZEN by using water:CAN:MeOH (90:5:5 for DON, or 43:35:22 for ZEN). Samples were separated isocratically with a flow rate of 0.3 mL/min for DON and ZEN. The photodiode array and fluorescence detector were used for DON and ZEN, respectively. The Waters Acquity UPLC^®^ BEH C_18_ column (2.1 × 100 mm, 1.7 μm particle size) was used for both toxins analyses. The MS system was run in the most appropriate ionization methods of ESI+ and ESI−. Quantitative analysis was carried out using a photodiode array (for DON) and fluorescence detector (for ZEN) with excitation and emission at 274 and 440 nm, respectively. The limit of detection (LOD) was 5 µg/kg for both DON and ZEN, and the limit of quantification (LOQ) was 10 µg/kg for DON and 16.7 µg/kg for ZEN. Along with standard diet, DON and ZEN contaminated corn feeds analysis was repeated three times. We found that the amount of DON and ZEN in the mixed corn feed was close to the original concentrations (7.38 mg DON/kg feed and 0.67 mg ZEN/kg feed). There was no DON and ZEN contamination in the control feed sample. 

### 5.4. Growth Performance

The BW of growing pigs was measured at day 0 and 28. BW and feed consumption data were used to calculate ADG, ADFI, and FCR per kg of BW. FCR is calculated as follows: FCR = feed intake/average daily gain.

### 5.5. Blood Collection

Blood samples were collected after four weeks by jugular venipuncture into collection tubes (BD Vacutainer^®^, Franklin Lakes, NJ, USA) containing ethylenediaminetetraacetic acid (EDTA) for hematological analysis, and in serum blood collection tubes (BD Vacutainer^®^, Franklin Lakes, NJ, USA) for immunological analysis (allowed to clot for 15–30 min). The serum was separated from the clot by centrifugation at 4 °C for 15 min at 2000 rpm. Hematological parameters were determined using an auto hematology analyzer (BC-5300Vet, Mindray, Hamburg, Germany). 

### 5.6. Tissues and Urine Collection

After four weeks of treatment, all pigs were killed by an anesthetic overdose, using pentobarbital. After cardiac arrest, the liver and kidneys were collected immediately and weighed. Muscle samples were also collected, rapidly frozen in liquid nitrogen, and stored at –80 °C for further analysis. After slaughtering the pigs, urine samples were collected directly from the urinary bladder using syringe. 

### 5.7. Histopathological Examination

Tissue samples from liver, kidney duodenum, jejunum, ileum, caecum, and colon were fixed in 10% formalin, and routinely processed and embedded in paraffin [52]. Tissues sections (5 µm) were cut using a microtome and stained with hematoxylin and eosin (H&E). Histological examination was performed under a light microscope by a pathologist. 

### 5.8. Measurement of Total Immunoglobulin Subsets 

Serum levels of IgG, IgA, and IgM were measured in the serum by enzyme-linked immunosorbent assay (ELISA), using commercial kits (IgA E101-102, IgG E101-104, and IgM E101-117, Bethyl Laboratories, Montgomery, TX, USA) as recommended by the manufacturer. The assays were conducted in 96-well, high binding microtiter plates (NUNC-Immuno Plate, VWR International, Batavia, IL, USA). The serum was diluted in 1× dilution buffer B (Tris-buffered saline, 0.05 M; Tween 20, 0.05%; bovine serum albumin [BSA], 1%) to 1:500,000, 1:10,000, and 1:50,000 for IgG, IgA, and IgM, respectively. The enzyme-substrate reaction was stopped by addition of 1 N H_2_SO_4_. Absorbance was read at 450 nm using an ELISA microplate reader (Versa Max, Sunnyvale, CA, USA). Immunoglobulins were quantified by reference to standard curves constructed with known amounts of pig immunoglobulin subsets. The detection limits were 12.5 ng/mL for IgA and IgG, and 10 ng/mL for IgM. The intra-assay coefficients of variability (CVs) were 16.18%, 13.70%, and 14.40% for IgA, IgG, and IgM, respectively. 

### 5.9. Measurement of TAC Levels

TAC measurement of biological fluids and other samples gives an indication of the overall ability to counteract reactive oxygen species (ROS), resist oxidative damage, and battle oxidative stress-related diseases. In the present study, TAC levels in serum and urine were measured by using a TAC kit (ab65329, Abcam, Cambridge, UK) according to the manufacturer’s instructions. Briefly, serum and urine samples were added in duplicate to 96-well plates and allowed to reduce Cu^2+^ in an orbital shaker for 1.5 h at room temperature. Here, the TAC assay uses Cu^++^ to Cu^+^ as a mechanistic tool, and this assay is strongly reducing, so some molecules, such as uric acid, will respond rapidly. Reduced Cu^++^ was chelated with a colorimetric probe and absorbances were measured at OD 570 nm in the Versa Max reader. Results are expressed as trolox equivalents according to a trolox standard curve (4–20 nmol trolox).

### 5.10. Measurement of Serotonin Levels

Serotonin levels were measured in serum and urine samples by ELISA using a commercial serotonin kit (ab133053; Abcam, Cambridge, MA, USA) according to the manufacturer’s recommendations. Serum and urine samples were diluted 1:16 ratio by using assay buffer. Samples and standards were added in duplicate to 96-well ELISA plates and results are reported as average values. A *P*-value lower than 0.05 indicated significance of the data.

### 5.11. RNA Isolation, Reverse Transcription, and Quantitative PCR (qPCR) 

Total RNA from liver, kidney, and muscle samples (0.3 g) was extracted using TRIzol^®^ Reagent (Ambion, Carlsbad, CA, USA) following the manufacturer’s instructions. Total RNA (3 µg) was used as template for cDNA synthesis using the GoTaq^®^ 2-step RT-qPCR system kit (Promega, Madison, WI, USA) with a final reaction volume of 20 µL. Synthesized cDNA was used as a template for qPCR to quantify the mRNA expression levels of various genes. Our aim was to obtain better insight into the changes produced by these mycotoxins in the expression of some marker genes (inflammatory cytokines and chemokines) accountable for important defense processes in the muscle, liver, and kidney organs. Primers were designed based on available pig (*Sus scrofa*) sequences using the Integrated DNA Technologies (IDT) Oligo Analyzer Tool. The primers sequences are indicated in Table 6. qPCR was carried out using a GoTaq^®^ qPCR master mix (Promega) and performed in a 7500 Fast Real-Time PCR Cycler (Applied Biosystems, Carlsbad, CA, USA). Reactions were run using the following PCR program: 95 °C for 5 min, 40 cycles of 95 °C for 15 s, 56 °C for 30 s, and 72 °C for 30 s, followed by a melting curve. Glyceraldehyde-3-phosphate dehydrogenase (GAPDH) was used as endogenous control for normalization. Relative abundance between treatment and control groups was calculated using the 2^−ΔΔCt^ method.

### 5.12. Statistical Analyses

Statistical analyses were performed using ANOVA (XLSTAT software, Version 2016.07, Addinsoft, Paris, France), followed by Duncan’s multiple-range test when appropriate. Differences between treatment groups were considered significant when *p* < 0.05. Results are presented as mean and SEM (standard error of mean).

## Figures and Tables

**Table 1:** Growth performance and relationship between organ-to-body weight ratio in pigs fed experimental diets containing deoxynivalenol (8 mg/kg) and zearalenone (0.8 mg/kg) mixed corn feed or a control diet.

Item	Control	DON	ZEN	SEM	*p*-Value
Initial BW (kg)	19.00	19.60	19.16	0.70	0.822
Final BW (kg)	43.50 ^a^	36.72 ^b^	42.68 ^a^	1.25	0.005
ADG (kg/day)	0.845 ^a^	0.590 ^b^	0.811 ^a^	0.03	0.0001
ADFI (kg/day)	1.41	1.31	1.38	0.07	0.536
FCR kg/kg	1.68 ^b^	2.25 ^a^	1.70 ^b^	0.11	0.005
Liver ratio (%)	3.22	3.42	3.49	0.18	0.501
Kidneys ratio (%)	1.22	1.30	1.31	0.08	0.761

Abbreviations: ADFI, average daily feed intake; ADG, average daily gain; BW, body weight; FCR, feed conversion rate; SEM, standard error of the mean. ^a, b^ Values with different letters within the same row are different (*p* < 0.05).

**Table 2:** Hematological values in pigs fed diets containing deoxynivalenol (8 mg/kg) and zearalenone (0.8 mg/kg) or a control diet.

Item	Control	DON	ZEN	SEM	*p*-Value
WBC	18.43	18.87	19.52	1.195	0.815
RBC	7.04	6.70	6.59	0.284	0.555
HGB	12.40	11.42	11.60	0.364	0.196
HCT	38.70	36.16	36.60	1.445	0.464
MCV	55.03	54.04	55.48	0.998	0.571
MCH	17.63	17.06	17.64	0.334	0.395
MCHC	32.08	31.62	31.80	0.404	0.738
RDW-CV	16.45 ^b^	18.42 ^a^	16.48 ^b^	0.417	0.008
RDW-SD	37.90 ^b^	41.66 ^a^	38.38 ^b^	0.811	0.013
PLT	187.25	225.20	330.40	49.147	0.147
MPV	7.58	7.58	7.54	0.23	0.991
PDW	16.05	15.86	15.96	0.181	0.771
PCT	0.14	0.17	0.25	0.037	0.155

Abbreviations: HCT, hematocrit; HGB, hemoglobin; MCH, mean corpuscular hemoglobin; MCHC, mean corpuscular hemoglobin concentration; MCV, mean corpuscular volume; PDW, platelet distribution width; PCT, procalcitonin; PLT, platelets; MPV, mean plate volume; RBC, red blood cells; RDW-CV, red blood cell distribution width-coefficient of variation; RDW-SD; red blood cell distribution width-standard deviation; SEM, standard error of the mean; WBC, white blood cells. ^a, b^ Values with different letters within the same row are different (*p* < 0.05).

**Table 3:** Immunological values, total antioxidant content, and serotonin values in pigs fed diets containing deoxynivalenol (8 mg/kg) and zearalenone (0.8 mg/kg) or a control diet.

Item		Control	DON	ZEN	SEM	*p*-Value
IgA (mg/mL)		0.61	0.86	0.69	0.12	0.456
IgG (mg/mL)		8.46 ^a^	5.97 ^c^	6.72 ^b^	0.15	0.0001
IgM (mg/mL)		1.84 ^a^	1.08 ^c^	1.24 ^b^	0.03	0.0001
Serum	TAC (nmol)	109.45 ^a^	88.24 ^b^	95.98 ^ab^	5.56	0.045
	Serotonin (μg/mL)	0.57	0.38	0.35	0.07	0.110
Urine	TAC (nmol)	2.36 ^b^	4.33 ^a^	2.40 ^b^	0.39	0.007
	Serotonin (μg/mL)	0.08 ^b^	0.17 ^a^	0.16 ^a^	0.02	0.020

Abbreviations: HCT, hematocrit; HGB, hemoglobin; MCH, mean corpuscular hemoglobin; MCHC, mean corpuscular hemoglobin concentration; MCV, mean corpuscular volume; PDW, platelet distribution width; PCT, procalcitonin; PLT, platelets; MPV, mean plate volume; RBC, red blood cells; RDW-CV, red blood cell distribution width-coefficient of variation; RDW-SD; red blood cell distribution width-standard deviation; SEM, standard error of the mean; WBC, white blood cells. ^a, b^^, ab, c^ Values with different letters within the same row are different (*p* < 0.05).

**Table 4:** Effect of deoxynivalenol (8 mg/kg) and zearalenone (0.8 mg/kg) exposure on the muscle, liver, and kidney expression of inflammatory cytokines.

Tissue	Inflammatory Genes	Dietary Treatments			SEM	Pr > F
		Control	DON	ZEN		
Muscle	IFN-γ	1.09 ^b^	2.01 ^a^	0.91 ^b^	0.22	0.009
	IL6	1.04 ^b^	1.89 ^a^	0.50 ^b^	0.24	0.004
	IL10	2.67 ^a^	1.28 ^b^	1.56 ^b^	0.27	0.011
	IL12B	4.60 ^ab^	6.11 ^a^	2.85 ^b^	0.78	0.032
	TNF-α	2.46 ^ab^	2.76 ^a^	1.87 ^b^	0.27	0.094
	PTGS2	5.15 ^a^	5.65 ^a^	2.43 ^b^	0.60	0.005
	CCL4	0.34	0.33	0.21	0.06	0.242
	CCL2	0.04 ^a^	0.03 ^ab^	0.005 ^b^	0.01	0.103
	CXCL10	3.42	2.93	1.90	1.69	0.813
	CLDN3	0.002	0.004	0.001	0.01	0.121
Liver	IFN-γ	4.43 ^ab^	3.94 ^a^	2.78 ^b^	1.02	0.036
	IL6	2.22	2.22	1.82	0.38	0.637
	IL10	2.67 ^a^	1.95 ^ab^	1.28 ^b^	0.31	0.029
	IL12B	3.79 ^a^	3.49 ^a^	1.93 ^b^	0.49	0.045
	TNF-α	2.38	2.36	1.22	0.37	0.076
	PTGS2	3.50	3.09	2.65	0.57	0.595
	CCL4	0.17 ^a^	0.05 ^b^	0.07 ^b^	0.02	0.007
	CCL2	0.17	0.09	0.16	0.03	0.168
	CXCL10	2.22	1.02	0.95	0.61	0.295
	CLDN3	0.32 ^a^	0.10 ^b^	0.11 ^b^	0.05	0.025
Kidney	IFN-γ	1.84	1.75	1.84	0.38	0.684
	IL6	5.29 ^a^	2.48 ^b^	3.74 ^ab^	0.82	0.102
	IL10	1.90	1.55	1.66	0.35	0.787
	IL12B	3.08	5.31	3.06	0.90	0.161
	TNF-α	2.20	2.02	1.81	0.32	0.699
	PTGS2	2.48	1.68	2.61	0.65	0.545
	CCL4	0.01 ^a^	0.006 ^b^	0.005 ^b^	0.00	0.028
	CCL2	0.12	0.06	0.06	0.03	0.285
	CXCL10	0.28	0.25	0.25	0.07	0.932
	CLDN3	0.01 ^a^	0.005 ^b^	0.007 ^ab^	0.01	0.083

Abbreviations: CCL, chemokine (C–C motif) ligand; CLDN3, claudin 3; CXCL, C–X–C motif chemokine ligand; IFNG, interferon gamma; IL, interleukin; PTGS2: prostaglandin-endoperoxide synthase 2; SEM, standard error of the mean; TNF-α: tumor necrosis factor α. ^a, b, ab^ Values with different letters within the same row are different (*p* < 0.05).

**Table 5:** Ingredients and chemical compositions of pig standard diet (as-fed basis).

Item	Control Diet
Ingredients (%)	
Ground corn	58.56
Soybean meal (46% crude protein)	14.00
Extruded soybean meal	12.00
Whey powder (12% crude protein)	7.00
Fish meal	3.45
Soybean oil	1.60
l-Lysine·HCL (78%)	0.43
dl-Methionine (99%)	0.14
l-Threonine (99%)	0.12
Calcium hydrophosphate	1.08
Limestone	0.60
Choline chloride (50%)	0.20
Sodium chloride	0.32
Vitamin–trace mineral premix ^a^	0.50
Calculated nutrients (%)	
Metabolizable energy (kcal/kg)	3444
Crude fiber	2.29
Crude protein	20.78
Lysine	1.47
Methionine	0.49
Calcium	0.75
Phosphorus	0.45

Notes: ^a^ Provided the following quantities per kg of complete diet: vitamin, A 11,000 IU; vitamin D3, 1500 IU; vitamin E, 44.1 IU; vitamin K3, 4.0 mg; vitamin B1, 1.4 mg; vitamin B2, 5.22 mg; vitamin B5, 20.0 mg; vitamin B12, 0.01 mg; niacin, 26.0 mg; pantothenic acid, 14 mg; folic acid, 0.8 mg; biotin, 44 mg; Fe, 100.0 mg as iron sulfate; Cu, 16.50 mg as copper sulfate; Zn, 90.0 mg as zinc sulfate; Mn, 35.0 mg as manganese sulfate; I, 0.30 mg as calcium iodate.

**Table 6:** Primer sequences used in the quantitative PCR assays.

Gene Name	Accession Number	Sequence 5′ to 3′	Product Size (bp)
IFN-γ	NM_213948	F: GGTAGCTCTGGGAAACTGAATG R: CTGACTTCTCTTCCGCTTTCTT	150
IL6	NM_214399	F: AGACGGATGCTTCCAATCTG R: CAGCCTCGACATTTCCCTTAT	134
IL10	NM_214041	F: ATCAAGGAGCACGTGAACTC R: CCTCTCTTGGAGCTTGCTAAA	144
IL12B	NM_214013	F: GAAGTACAGAGTGGAGTGTCAG R: TGATGAAGAAGCTGCTGGTATAG	128
TNF-α	NM_214022	F: CCTACTGCACTTCGAGGTTATC R: GGCTTTGACATTGGCTACAAC	145
PTGS2	NM_214321	F: GATGGCCACGAGTACAACTATC R: AAGATTCCTACCACCAGCAAC	129
CLDN3	NM_001160075	F: TCATCGGCAGCAGCATTATC R: GAGAGTCGTACACTTTGCACTG	105
CXCL10	NM_001008691	F: CAAGGAATACCTCTCTCCAGAAC R: ATCTCAACATGTGGGCAAGA	128
CCL4	NM_213779	F: TCCTGCTGCTTCACATACAC R: TACTCCTGGACCCAGTCATC	158
CCL2	NM_214214	F: TCACCTGCTGCTATACACTTAC R: GGTTCTGCACAGATCTCCTT	139
GAPDH	AF017079	F: GTCTGGAGAAACCTGCCAAATA R: CCCAGCATCAAAGGTAGAAGAG	152

Abbreviations: CCL: chemokine (C–C motif) ligand; CLDN3: claudin 3; CXCL10: C–X–C motif chemokine ligand 10; F: forward primer, GAPDH: glyceraldehyde 3-phosphate; IFN-γ: interferon gamma; IL: interleukin; TNF-α: tumor necrosis factor; PTGS2: prostaglandin-endoperoxide synthase 2; R: reverse primer.
